# Appendiceal agenesis in a 3-year-old boy presenting with suspected acute appendicitis: a case report

**DOI:** 10.3389/fped.2026.2012352

**Published:** 2026-10-21

**Authors:** Xiaofeng Yang, Lijun Wang, Dan Zhang, Xinhong Yang

**Affiliations:** 1 Department of Pediatric Surgery, the Affiliated Hospital of Chengde Medical College, Chengde, Hebei, China; 2 Chengde Nursing Vocational College, Chengde, Hebei, China; 3 Department of Hematology Laboratory, the Affiliated Hospital of Chengde Medical College, Chengde, Hebei, China

**Keywords:** acute appendicitis, appendiceal agenesis, appendix, ileocecal, lymphadenopathy

## Abstract

Appendiceal agenesis is a rare congenital gastrointestinal anomaly, usually discovered unexpectedly during surgery for suspected acute appendicitis. It cannot be diagnosed pre-operatively by clinical, laboratory or imaging findings, and final confirmation requires thorough surgical inspection of the ileocecal region. We report a 3-year-old boy with 38-hour intermittent abdominal pain, bilious vomiting and fever suggestive of acute appendicitis. Pre-operative CT failed to visualize the appendix but showed pericecal inflammation and enlarged mesenteric lymph nodes. Laparoscopy combined with limited open laparotomy confirmed Collins Type-III appendiceal agenesis (complete absence of the appendix with an intact caecum), accompanied by localized peritonitis and intra-abdominal adhesions. Mesenteric lymph node biopsy revealed reactive lymphadenopathy, excluding granulomatous or neoplastic lesions. The child had an uneventful postoperative recovery and was discharged in satisfactory condition. This case highlights that appendiceal agenesis should be included in the differential diagnosis when the appendix cannot be located intra-operatively, and surgeons should adhere to standardized exploration protocols before establishing this diagnosis.

## 1 Introduction

Since Morgagni first documented this entity in 1718, congenital absence of the vermiform appendix has remained a seldom-encountered developmental defect. Its estimated incidence is roughly one in 100,000 surgical explorations performed for suspected appendicitis, with an autopsy detection rate of 0.006%) (1). Embryologically, the vermiform appendix evolves from a caecal diverticulum at the eighth week of intrauterine development. Disrupted embryogenesis at this stage can result in complete appendiceal agenesis (2). Potential contributing factors include intrauterine ischaemic injury, teratogenic influences such as maternal thalidomide exposure, and rare intrauterine spontaneous auto-amputation of the appendix (3).

Collins proposed a five-tiered classification framework for caeco-appendiceal developmental defects. Among these categories, Type-III (normal-looking caecum without appendix) accounts for the largest proportion of clinical cases (4). We report a paediatric patient whose clinical picture strongly suggested acute appendicitis, and who was finally diagnosed with Collins Type-III appendiceal agenesis intra-operatively.

## 2 Case presentation

A previously healthy 3-year-1-month-old Chinese male child was admitted to the hospital department for intermittent abdominal pain lasting 38 h together with fever, and intermittent fever peaked at 38.0 °C. The child accompanied by 7-8 episodes of non-projectile bilious vomiting. Ten hours prior to hospital presentation, abdominal pain worsened and shifted to the right abdomen. The child denied diarrhoea and dysuria, and had not passed stool. The child had no history of prior surgery, trauma or blood transfusion. He was born preterm at 36 + 4 weeks of gestation via caesarean delivery without perinatal asphyxia. His immunization schedule was complete, with no known drug or food allergies.

Abdominal assessment revealed moderate abdominal distension, marked right-sided abdominal tenderness with guarding and rebound tenderness. The liver and spleen were non-palpable; percussion yielded tympanic note, and bowel sounds decreased to 1-2 times per minute. No surgical abdominal scars were identified.

Laboratory work-up showed leukocytosis with neutrophil predominance (WBC was 12.9 × 10^9^/L and neutrophil counts were 9.82 × 10^9^/L), alongside elevated C-reactive protein (45.75 mg/L). Haemoglobin and platelet values fell within paediatric reference ranges. Abdominal-pelvic CT demonstrated non-visualization of the appendix, pericecal exudative changes and multiple enlarged mesenteric lymph nodes. Pre-operative clinical impression was acute abdomen, highly suggestive of acute appendicitis with localized peritonitis is shown in Figure 1.

**Figure 1:**
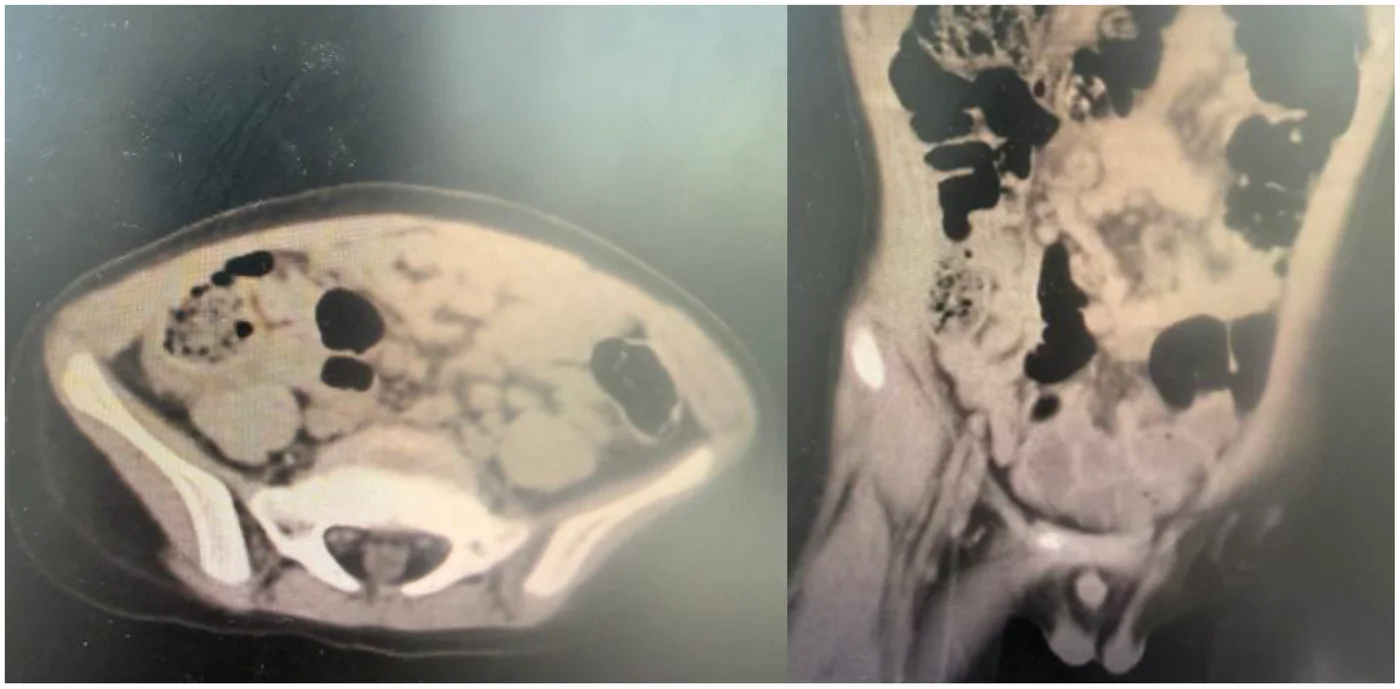
Abdominal-pelvic CT scan. No appendix can be visualized; pericecal inflammatory exudation and enlarged mesenteric lymph nodes are observed.

He was taken for a laparoscopic appendectomy. The caecum was positioned in the right mid-abdomen, with adhesions attaching it to the lateral abdominal wall and right iliac fossa. After tracing taeniae coli towards their junction, neither appendix nor appendiceal remnant could be identified. Blunt and sharp lysis of adhesions was performed; the retrocaecal peritoneum was incised for full exposure of the retrocaecal space, still without identification of appendiceal tissue. A 2.5 cm extension of the abdominal incision allowed exteriorization of the ileocaecal segment for direct manual palpation of the caecal wall are shown in Figure 2. No intramural or intussuscepted appendiceal tissue could be detected, consistent with Collins Type-III appendiceal agenesis. Markedly enlarged mesenteric lymph nodes (maximum dimension 2.5 × 2.5 × 3 cm) were observed. Approximately 1.5 metres of distal small bowel were inspected in retrograde fashion starting from the ileocaecal junction, excluding Meckel's diverticulum and other small-bowel malformations. Two representative lymph node specimens (including the largest node) were harvested for biopsy. Specimens were submitted for routine postoperative formalin-fixed paraffin-embedded histopathological examination; intraoperative frozen-section analysis was not performed. Post-operatively, intravenous cefoperazone-sulbactam and metronidazole were administered for anti-infective management, combined with fluid resuscitation and supportive care. Fever subsided gradually and abdominal discomfort resolved.

**Figure 2:**
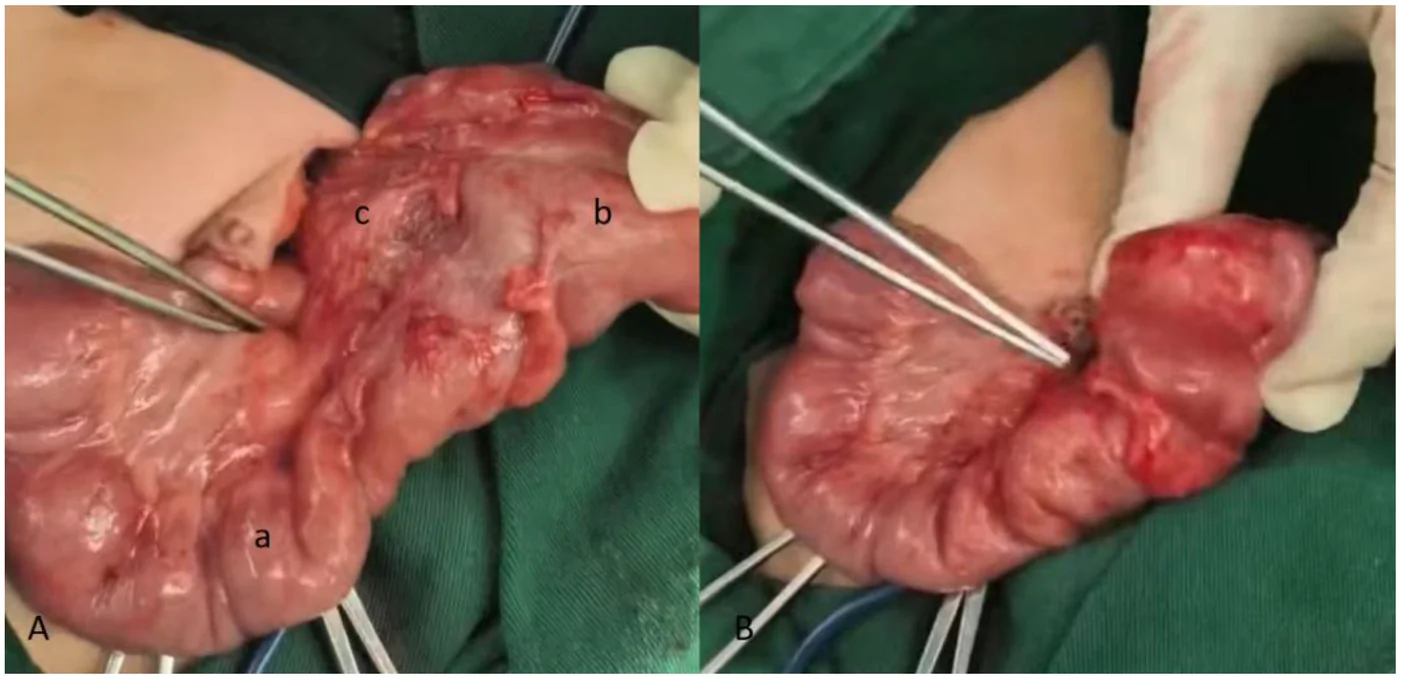
Intra-operative photographs of the ileocecal region. **(A)** Anterior view of the ileocecal region. a: ileum; b: cecum; c: ascending colon. **(B)** Posterior view of the ileocecal region; no appendiceal tissue is identified.

On postoperative day 3, the child achieved flatus and defecation, oral intake was initiated with clear fluids and gradually advanced to regular diet, and without vomiting or abdominal distension. Surgical incisions healed primarily. The patient was discharged on postoperative day 6 in stable clinical condition, with final lymph-node histopathology still pending at discharge. The patient was followed-up for 3 months after discharge. He remained asymptomatic, with no recurrent abdominal pain or fever; abdominal physical examination was unremarkable. The final permanent histopathological report confirmed reactive lymphadenopathy; no granulomatous inflammation or neoplastic lesions were identified.

## 3 Discussion

Appendiceal agenesis poses diagnostic challenges for paediatric surgeons. Affected patients frequently exhibit clinical manifestations mimicking acute appendicitis, including right-sided abdominal pain, peritoneal irritation signs, elevated inflammatory biomarkers, and non-specific pericecal inflammatory changes on cross-sectional imaging without clear appendix visualization (5). It should be emphasized that failure to visualize the appendix on CT scan is non-specific. This radiological finding may result from appendiceal inflammation, retrocaecal anatomical position or imaging technical limitations, and cannot independently establish congenital appendiceal absence.

Multiple differential diagnoses should be ruled out before confirming appendiceal agenesis intra-operatively. These include prior appendectomy (excluded in this patient given negative surgical history), intramural appendix, appendiceal intussusception, appendiceal atresia, hypoplastic appendix (Collins Type-IV) and ectopic appendix located in rare intra-abdominal sites (6). Ectopic appendix can be located in several unusual sites, including retrohepatic/subhepatic space, ascending-colon wall, thoracic cavity (secondary to diaphragmatic hernia), and even deep gluteal space via greater sciatic foramen; retrocaecal and pelvic positions represent the most common anatomical variants, whereas those above are extremely rare ectopic sites (7).

The exact pathogenetic mechanism for appendiceal agenesis and appendiceal atresia remains incompletely understood. The most widely-accepted hypothesis is intrauterine mesenteric vascular accident occurring during 7-12 weeks of gestation, leading to ischemic injury of the caecal diverticulum primordium. Additional proposed contributing factors include teratogenic exposure such as maternal thalidomide intake with anti-angiogenic effects; intrauterine spontaneous auto-amputation of appendix secondary to in-utero torsion or inflammation can mimic congenital agenesis, which should be distinguished from true developmental defect. Appendiceal atresia represents a related but distinct developmental entity: the appendiceal lumen is discontinuously obliterated while appendiceal tissue persists, and it may present clinically as acute suppurative appendicitis despite luminal discontinuity from caecum, as described by Yaylak et al. in 2013. Appendiceal agenesis may appear as an isolated anomaly, or it may co-exist with other congenital gastrointestinal malformations including omphalocele, ileocecal atresia, congenital short-bowel syndrome, and cloacal malformations, suggesting shared early embryonic insults affecting midgut-derived structures (8).

Standardized surgical evaluation consists of confirming surgical background, full mobilization of the caecum and ascending colon, following taeniae coli to their convergence point, thorough exploration of retrocaecal and ileocaecal zones, manual palpation of the caecal wall, exclusion of Meckel's diverticulum, and identification of alternative sources for acute abdomen such as mesenteric lymphadenitis (9). In this case, exteriorization of the ileocaecal segment allowed direct palpation, eliminating the possibility of wall-embedded appendix.

Collins classification system divides ceco-appendiceal developmental anomalies into five subtypes: Type I: Complete absence of both caecum and appendix; Type II: Rudimentary caecum with absent appendix; Type III: Normal-appearing caecum with complete absence of appendix (our patient belonged to this most-common subtype); Type IV: Normal caecum with rudimentary/hypoplastic appendix; Type V: Giant dilated caecum without appendix. This patient corresponded to Type-III agenesis, which represents the most prevalent phenotype reported in available literature. Appendiceal agenesis may occur in isolation, or co-exist with a spectrum of other congenital gastrointestinal anomalies including omphalocele, ileocaecal atresia, short-bowel syndrome, ileal duplication and cloacal malformations (9, 10).

No additional intestinal developmental defects were discovered during exploration in our patient. The peritonitis-like presentation was most likely secondary to intra-abdominal adhesions and reactive mesenteric lymphadenopathy. Biopsy of markedly enlarged lymph nodes was performed to exclude infectious or neoplastic aetiologies, which constituted an essential diagnostic step.

Once appendiceal agenesis is reliably confirmed intra-operatively, clinical attention should shift toward identifying alternative sources responsible for the acute-abdomen picture. Isolated appendiceal agenesis carries an excellent long-term prognosis, as congenital absence of the appendix does not produce known persistent physiological impairment (11).

## 4 Conclusion

Appendiceal agenesis is a rare but noteworthy differential diagnosis in paediatric patients with clinical suspicion of acute appendicitis. Pre-operative laboratory investigations and imaging modalities are incapable of establishing this diagnosis. Strict adherence to standardized intraoperative exploration protocols is mandatory before confirming appendiceal agenesis, so as not to overlook intramural, intussuscepted or ectopic appendix. After confirming congenital absence of the appendix, thorough assessment for alternative causes of acute abdominal pain remains essential.

## Data Availability

The raw data supporting the conclusions of this article will be made available by the authors, without undue reservation.

## References

[1] Greenberg SL Eyers AA Mackay S. Congenital absence of the vermiform appendix. ANZ J Surg. (2003) 73:166–7. 10.1046/j.1445-2197.2003.02642.x 12608987

[2] Yohannes B Abebe K. Appendiceal agenesis: a very rare intraoperative diagnosis—a case report. Int Med Case Rep J. (2021) 14:233–6. 10.2147/IMCRJ.S309192 33859503 PMC8043782

[3] Shand JE Bremner DN. Agenesis of the vermiform appendix in a thalidomide child. Br J Surg. (1977) 64:203–4. 10.1002/bjs.1800640316 890268

[4] Chèvre F Gillet M Vuilleumier H. Agenesis of the vermiform appendix. Surg Laparosc Endosc Percutan Tech. (2000) 10:110–2. 10.1097/00129689-200004000-00014 10789585

[5] Hammood ZD Salih AM Mahal LA Yas YT Ghaleb HA Kakamad FH. Agenesis of vermiform appendix; a case report with literature review. Int J Surg Case Rep. (2021) 87:106364. 10.1016/j.ijscr.2021.106364 34562720 PMC8473660

[6] Yaylak F Algin MC Gungor Y Korkmaz M. Atresia of the appendix vermiformis: a rare case of developmental abnormality. World J Gastroenterol. (2013) 19:122–4. 10.3748/wjg.v19.i1.122 23326173 PMC3542758

[7] Ikidag MA. Ectopic appendix vermiformis located in the right deep gluteal region due to unilateral piriformis agenesis. Surg Radiol Anat. (2019) 41:141–2. 10.1007/s00276-018-2125-y 30361841

[8] Vincent MV Doyle A Bernstein S Jackman S. Absence of the appendix discovered during childhood. Springerplus. (2014) 3:522. 10.1186/2193-1801-3-522 25279313 PMC4166596

[9] Zetina-Mejía CA Alvarez-Cosío JE Quillo-Olvera J. Congenital absence of the cecal appendix. Case Report. Cir Cir. (2009) 77:407–10. 19944032

[10] Mellouki MT Benmassaoud Z Zouirech Y Cherrabi H. Rare association of type 1 omphalocele with ileocecal atresia and congenital appendiceal agenesis: a case report. Cureus. (2026) 18:e110746. 10.7759/cureus.110746 42438628 PMC13356905

[11] Malik F Arif M Irshad A Nassar H Arshad A Adeel M. Suspected congenital absence of the vermiform appendix in a 13-year-old girl: a case report. Cureus. (2026) 18:e113918. 42694634 10.7759/cureus.113918 PMC13539338

